# Cytosolic RNA binding of the mitochondrial TCA cycle enzyme malate dehydrogenase

**DOI:** 10.1261/rna.079925.123

**Published:** 2024-07

**Authors:** Michelle Noble, Aindrila Chatterjee, Thileepan Sekaran, Thomas Schwarzl, Matthias W. Hentze

**Affiliations:** European Molecular Biology Laboratory (EMBL), Heidelberg 69117, Germany

**Keywords:** RNA-binding proteins, MDH2, metabolic enzymes

## Abstract

Several enzymes of intermediary metabolism have been identified to bind RNA in cells, with potential consequences for the bound RNAs and/or the enzyme. In this study, we investigate the RNA-binding activity of the mitochondrial enzyme malate dehydrogenase 2 (MDH2), which functions in the tricarboxylic acid (TCA) cycle and the malate–aspartate shuttle. We confirmed *in cellulo* RNA binding of MDH2 using orthogonal biochemical assays and performed enhanced cross-linking and immunoprecipitation (eCLIP) to identify the cellular RNAs associated with endogenous MDH2. Surprisingly, MDH2 preferentially binds cytosolic over mitochondrial RNAs, although the latter are abundant in the milieu of the mature protein. Subcellular fractionation followed by RNA-binding assays revealed that MDH2–RNA interactions occur predominantly outside of mitochondria. We also found that a cytosolically retained N-terminal deletion mutant of MDH2 is competent to bind RNA, indicating that mitochondrial targeting is dispensable for MDH2–RNA interactions. MDH2 RNA binding increased when cellular NAD^+^ levels (MDH2's cofactor) were pharmacologically diminished, suggesting that the metabolic state of cells affects RNA binding. Taken together, our data implicate an as yet unidentified function of MDH2-binding RNA in the cytosol.

## INTRODUCTION

During the past decade, numerous metabolic enzymes at the heart of intermediary metabolism have emerged to bind RNA (Cieśla 2006; Baltz et al. 2012; Castello et al. 2012; Hentze et al. 2018). Several metabolic enzymes (e.g., ACO1, GAPDH, TYMS, IMPDH, PKM2) have been found to moonlight in posttranscriptional regulatory functions (Casey et al. 1988; Chu et al. 1991; Hentze and Argos 1991; Constable et al. 1992; Singh and Green 1993; Nagy and Rigby 1995; Dollenmaier and Weitz 2003; Zhou et al. 2008; Hentze and Preiss 2010; McGrew and Hedstrom 2012; Kejiou et al. 2023). In contrast, the enzymatic activities of ENO1 and SHMT1 have been found to be riboregulated by cytosolic RNAs (Guiducci et al. 2019; Huppertz et al. 2022). Although these examples underline physiologically significant roles of metabolic enzyme–RNA interactions, these cannot be assumed on the basis of binding data alone, and the roles of most metabolic enzyme–RNA interactions await detailed exploration.

Malate dehydrogenase has repeatedly been identified to interact with RNA across biological model systems ranging from *Escherichia coli* to human cells. Its RNA binding has been detected in multiple large-scale RNA interactome studies (Scherrer et al. 2010; Baltz et al. 2012; Castello et al. 2012, 2016; Kwon et al. 2013; Beckmann et al. 2015; Matia-González et al. 2015; He et al. 2016; Liao et al. 2016; Marondedze et al. 2016; Wessels et al. 2016; Mullari et al. 2017; Bao et al. 2018; Huang et al. 2018; Perez-Perri et al. 2018, 2023; Shchepachev et al. 2018; Esmaillie et al. 2019; Garcia-Moreno et al. 2019; Mallam et al. 2019; Panhale et al. 2019; Queiroz et al. 2019; Trendel et al. 2019; Urdaneta et al. 2019; Backlund et al. 2020), purified together with poly(A) RNA as well as total RNA (Caudron-Herger et al. 2021; Gebauer et al. 2021; Schwarzl et al. 2022). Like most mitochondrial proteins, malate dehydrogenase 2 (MDH2) is encoded by the nuclear genome, synthesized as a precursor protein in the cytosol, and then imported into the mitochondrial matrix (Chien et al. 1984; Grant et al. 1986, 1987; Chu et al. 1987; Sztul et al. 1988), where it assembles into functional homodimers. Together with its cytosolic isoform MDH1, MDH2 forms part of the malate–aspartate shuttle, transporting reducing equivalents and metabolites across the mitochondrial membrane (Berg et al. 2002). It thus supports the preservation of the cellular redox state across compartments. Dysfunctions in MDH2 expression and activity are involved in several pathologies, including different forms of cancer and neurodevelopmental disorders (Liu et al. 2013; Cascón et al. 2015; Lo et al. 2015; Ait-El-Mkadem et al. 2017).

MDH2 belongs to the 2-hydroxy acid dehydrogenase superfamily and catalyzes the reversible conversion of malate to oxaloacetate with the simultaneous reduction of NAD^+^ to NADH. Like other dehydrogenases, MDH2 has an NAD^+^-binding Rossman fold, which has been proposed as an RNA-binding domain (Hentze 1994; Nagy et al. 2000; Castello et al. 2015; Liao et al. 2016). MDH2's RNA-binding function has been implicated in the posttranscriptional regulation of SCN1A mRNA by binding to its 3′ UTR (Chen et al. 2017). It has also been reported to promote tumorigenesis via lncRNA AC020978 (Xu et al. 2021). Additionally, MDH2 binding to lncRNA GAS5 was linked to tricarboxylic acid (TCA) cycle regulation by disrupting the formation of an MDH2–FH–CS metabolon complex (FH, fumarate hydratase; CS, citrate synthase) (Sang et al. 2021). Although these studies uncover possible functions of the RNA-binding activity of MDH2, they have focused on individual MDH2–RNA interactions, and an unbiased, comprehensive analysis of MDH2's RNA-binding properties is still lacking.

We, therefore, used eCLIP (Van Nostrand et al. 2016) to determine the scope of RNA binding by MDH2. In addition, we provide insights into the cellular locale of MDH2–RNA interactions as well as potential effects of MDH2's cofactor NAD^+^ on its apparent RNA-binding activity.

## RESULTS

### Human mitochondrial malate dehydrogenase binds RNA in Huh7 cells

**FIGURE 1.**
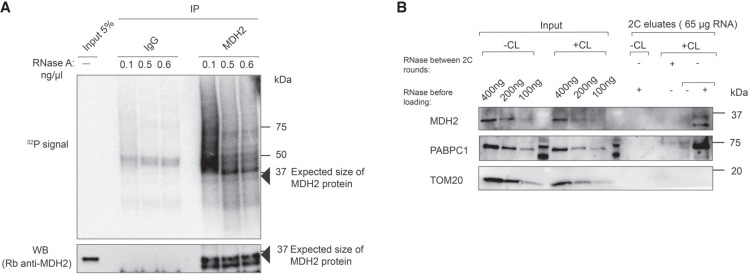
MDH2 binds RNA in cells. (*A*) RNA-binding activity of MDH2 in PNK assay. (*Top* panel) The phosphor image to visualize RNA copurified with MDH2 following γ-32P ATP labeling by PNK. (*Bottom* panel) A representative western blot to confirm equal IP efficiencies. Black arrows indicate the expected molecular mass of MDH2. (*B*) RNA-binding activity of MDH2 assessed by complex capture (2C) assay. Specific MDH2 western blot signals are detected when the sample has been treated with RNase before loading to visualize it around the expected molecular mass. RNase treatment before a second round of 2C abolishes the western blot signal. PABPC1 was used as a positive control and TOM20 as a negative control.

We probed the RNA binding of MDH2 in the human hepatocarcinoma cell line Huh7 using two orthogonal biochemical approaches. For the polynucleotide kinase (PNK) assay (Richardson 1965), cells were exposed to ultraviolet (UV) light (254 nm, 150 mJ/cm^2^) to induce covalent bonds between RNA-binding proteins and their bound RNAs, followed by MDH2 immunoprecipitation (IP) from cellular lysates. The 5′ ends of MDH2-associated RNAs were subsequently labeled in vitro with γ-^32^P ATP and assessed by denaturing gel electrophoresis. In the PNK assay for MDH2, RNase-sensitive autoradiography signals were detected after MDH2 IP. When the RNase digestion is limited, large MDH2–RNA complexes migrate as a smear on a gel, indicating diverse lengths of the RNAs bound to MDH2. Following more extensive RNase digestion, the signals condense to a band slightly above the expected molecular mass of native MDH2. An identical assay with isotype-matched IgG as a specificity control showed only minor background signals (Fig. 1A). Moreover, we also find that the RNA signal is dependent on UV cross-linking, further confirming its specificity (Supplemental Fig. S1).

The 2C assay identifies RNA binding based on RNA-mediated protein retention on silica columns that are routinely used for RNA purification (Asencio et al. 2018). Following UV cross-linking, samples eluted from the silica column are assessed for the presence of proteins of interest by western blotting. Before gel analysis, the eluates are digested with RNase to minimize the contribution of RNA to the molecular mass of the RNA–protein complex and to visualize the protein near its expected molecular mass. 2C reveals that MDH2 is retained on the silica column in a UV cross-linking–dependent way and requires RNA, because RNase treatment before a second round of 2C abolishes MDH2 retention. Canonical RNA-binding proteins like PABPC1 [poly(A)-binding protein] show a similar behavior, whereas TOM20 (without identified RNA-binding activity) used as a specificity control is not copurified (Fig. 1B).

### MDH2 binds predominantly cytosolic RNAs

Having confirmed cellular RNA binding of MDH2, we used enhanced cross-linking and immunoprecipitation (eCLIP) (Van Nostrand et al. 2016) to determine which RNAs are bound by MDH2. RNAs cross-linked to MDH2 were identified by sequencing after MDH2 IP from total cell lysates rather than lysates from purified mitochondria to avoid a data bias based on a priori assumptions about where MDH2 may bind RNA. We used comparison against a size-matched input (SMI) control to identify RNA targets bound by MDH2. This approach has been established to remove background noise originating from RNAs with a similar gel migration as the RNP complexes of interest (Van Nostrand et al. 2016). A control IP experiment performed with isotype-matched IgG was also used to determine binding specificity.

After cDNA preparation, we ensured the quality of the sequencing libraries by optimizing the number of PCR cycles required for library preparation in test PCRs (with 10% of the cDNA samples) followed by PAGE analysis. The final replicate libraries were pooled and assessed using their bioanalyzer profiles (Supplemental Fig. S2A). We generated three independent replicate sequencing libraries for MDH2 IP (∼9.1 million uniquely mapped reads/sample), SMI (∼4.3 million uniquely mapped reads/sample), and isotype-matched IgG IP (∼4.3 million uniquely mapped reads/sample). Principal component analysis confirmed close clustering of respective replicates distinct from the clusters of other samples (SMI/MDH2 IP/IgG IP) (Supplemental Fig. S2B).

**FIGURE 2.**
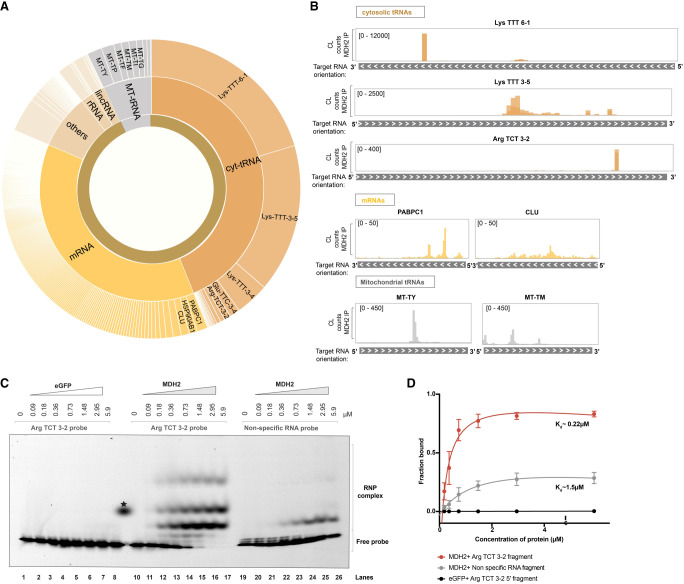
MDH2 predominantly binds cytosolic RNAs. (*A*) Sunburst chart showing the distribution of cross-linking counts in binding sites identified from MDH2 eCLIP data. “Cytosolic” indicates nuclear-encoded RNAs and “Mito” indicates mitochondrially transcribed RNAs. (*B*) Cross-linking (CL) count distribution of selected target RNAs bound by MDH2 visualized using the IGV interactive tool (Robinson et al. 2011). (*C*) Representative electromobility shift assay (EMSA) assays were performed to assess the affinity of MDH2 to a tRNA Arg TCT 3-2 probe or a control RNA probe (RPS23 5′-UTR sequence). Binding of eGFP to the target RNA probe is included as a negative control. (*D*) Binding affinities from EMSA experiments were estimated using a nonlinear regression fit (solid line). All shifted bands (all bands above the free migrating RNA) were included in the quantification of total RNA binding. Data are reported as mean ± SD (dots with error bars). Number of experiments, *n* = 3–4 from two independent protein purifications.

In total, we identified 524 distinct, specifically enriched binding regions for MDH2 (Supplemental Table S1) across 361 unique RNAs, a major proportion of them on RNAs expressed outside of mitochondria (Fig. 2A shows the distribution of cross-linking counts across MDH2 targets). This observation was unexpected, considering MDH2's primary enzymatic function and its enrichment in the mitochondrial matrix (Aziz et al. 1981; Sztul et al. 1988; Musrati et al. 1998). MDH2's unexpected preference for cytosolic RNAs (transcribed from nuclear genomic DNA) over the abundant MT-RNAs (transcribed from mitochondrial DNA) concentrated in the locale of the mature protein suggests that its RNA association may primarily be relevant in the cytosol.

Among the mitochondrial RNAs associated with MDH2, a preference of MDH2 for tRNAs emerged. MDH2 binds 9 of the 22 MT-tRNAs (Suzuki et al. 2011) expressed in human mitochondria (Supplemental Table S1). MDH2's propensity to bind tRNAs is also reflected among the cytosolic RNA interactors, with cytosolic tRNAs (specifically Lys TTT isoacceptor) (Fig. 2A,B; Supplemental Table S1) emerging as the top MDH2 binders in the eCLIP analysis. Arg TCT and Glu TTC tRNA isoacceptors also feature among the cytosolic tRNAs with enriched MDH2 cross-linking sites. In addition, MDH2 binds to several other classes of cytosolic RNAs including mRNAs, lincRNAs, rRNAs, and others (Fig. 2A). Highly abundant mRNAs like PABPC1 and CLU (Fig. 2B) come up as top hits among mRNAs. We also analyzed the distribution of cross-linking sites on MDH2's previously identified interactors (SCN1A, lncRNA AC020978, GAS5) (Chen et al. 2017; Sang et al. 2021; Xu et al. 2021) in our eCLIP data set. Cross-linking sites of MDH2 on SCN1A mRNA were not detected in our eCLIP experiment performed in Huh7 cells, which could be due to the predominantly brain-specific expression of this mRNA (Heighway et al. 2022). Although we detected cross-linking sites on the other previously identified MDH2 interactors, lncRNA AC020978 and GAS5, these were not enriched over the SMI control in Huh7 cells.

To assess the binding of MDH2 to a target RNA in vitro, we selected one of the tRNAs enriched in the MDH2 eCLIP data set, Arg TCT 3-2. Based on initial screening, A chemically synthesized 35-nt RNA probe matching a segment of Arg TCT 3-2 tRNA, tagged with a fluorescent Cy5 label at the 3′ end, was used to assess RNA binding. We also designed a control probe derived from the 5′ UTR of RPS23 mRNA (not enriched in the MDH2 eCLIP data set) to evaluate the specificity of MDH2–RNA interactions. Full-length human MDH2 including the N-terminal mitochondrial targeting signal (MTS) and corresponding to the cytosolic preprotein, was expressed and purified from the *E. coli*-Rosetta (DE3) strain (Supplemental Fig. S3A–C). A C-terminal Strep-II tag was used for protein purification and retained for the binding assays. eGFP protein, purified similarly and bearing the same tag (Supplemental Fig. S3A,D), served as a specificity control.

In vitro, EMSA confirmed MDH2's interaction (0.18–5.9 μM, lanes 11–17) with the Arg TCT 3-2 probe (Fig. 2C,D) that was fixed at 10 nM in all experiments. Comparison with the control RNA probe (lanes 19–26) and the lack of binding to eGFP (lanes 1–8) confirm the specificity of the interaction (Fig. 2C,D). By quantifying the fraction of RNA bound at increasing protein concentrations, we estimated MDH2's binding affinity to the Arg TCT 3-2 RNA probe (*K*_d_ = 0.22 μM) and to the control RNA (*K*_d_ = 1.5 μM) (Fig. 2D), suggesting that MDH2 can discriminate between different RNA fragments and confirming Arg TCT 3-2 tRNA as a preferred binding partner.

### MDH2 binds RNA preferentially outside of the mitochondria

**FIGURE 3.**
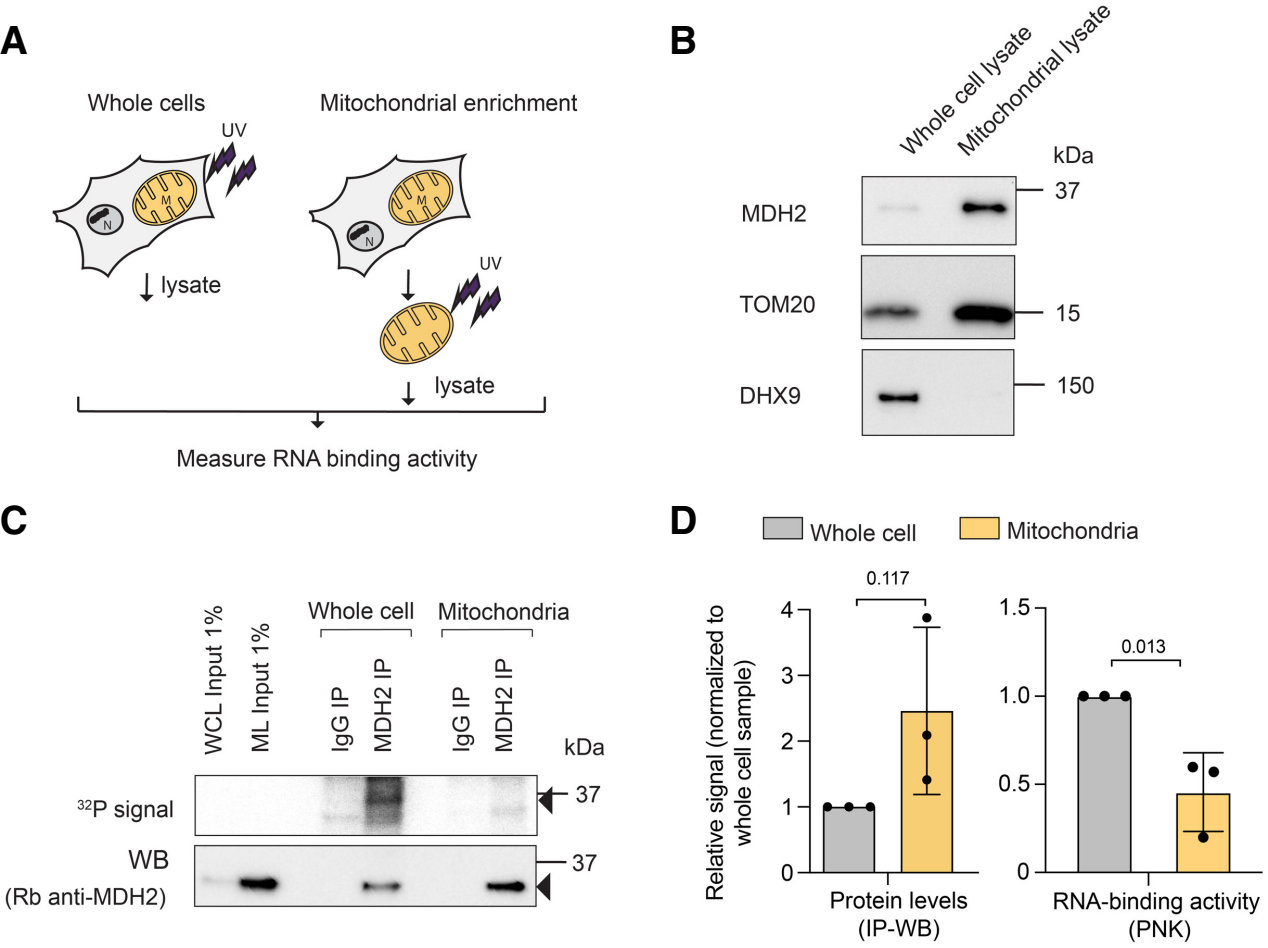
MDH2 binds RNA preferentially outside of mitochondria. (*A*) Experimental scheme. (*B*) Western blot confirming mitochondrial enrichment. TOM20, an outer mitochondrial protein, was used as a mitochondrial marker; DHX9, a cytosolic protein, was used to assess the purity of the mitochondrial preparation. (*C*) PNK assay for MDH2 reveals a stronger RNA signal from whole-cell lysates compared to mitochondrially enriched fractions. Black arrows indicate bands near the expected molecular mass of MDH2. (*D*) Quantification of immunoprecipitated MDH2 (IP-WB) and copurified RNA (IP-PNK). Data are reported as mean ± SD number of experiments, *n* = 3, individual *P*-values are indicated according to an unpaired two-tailed *t*-test.

The cytosolic MDH2 preprotein is imported into the mitochondrial matrix, where it is cleaved to form the mature MDH2 protein (Aziz et al. 1981). Considering that MDH2 associates with cytosolic RNAs, we wanted to determine the subcellular locale of MDH2's RNA binding. We performed comparative PNK assays on MDH2 purified from whole cell versus mitochondrially enriched fractions (Fig. 3A). The purity of the mitochondrial fractions was confirmed by enrichment of the outer mitochondrial membrane marker, TOM20, and the depletion of cytosolic proteins (DHX9) in the mitochondrial lysates (Fig. 3B). Consistent with MDH2's evident localization in the mitochondrial matrix, we observed higher MDH2 signals in the input and the MDH2-immunoprecipitated samples from the mitochondrially enriched cell fractions (Fig. 3C). Nonetheless, the PNK signal intensity for MDH2 was markedly lower from the mitochondrial fraction than from the whole-cell lysate (Fig. 3C,D). Thus, combined with MDH2's preference for cytosolic tRNAs, this result indicates that MDH2 binds RNA preferentially outside of mitochondria.

To further characterize the functional parameters of MDH2's RNA binding, we mutated MDH2 to be constrained to the cytosol. MDH2's MTS was surmised to correspond to the N-terminal 1–24 amino acids by sequence similarity to the MDH2 targeting signals from other organisms including rat (94.4% identity with the human MDH2) (Chien et al. 1984; Chu et al. 1987; Grant et al. 1987; Sztul et al. 1988, 1989), where extensive mitochondrial import studies had been performed. This region possesses the typical characteristics of an MTS, with basic, positively charged, and hydroxylated residues (Wiedemann and Pfanner 2017; Busch et al. 2023). Based on this information, we deleted this N-terminal region from MDH2 (MDH2 Δ2–24), and evaluated the effect of this deletion on MDH2 localization and RNA binding (Supplemental Fig. S4A).

To our surprise, the deletion of amino acids 2–24 did not confine MDH2 Δ2–24 to the cytosol (Supplemental Fig. S4D). Instead, the MDH2 Δ2–24 mutant protein displays costaining with the mitochondrial marker TOM70, indicating intact mitochondrial targeting. MDH2 Δ2–24 retained RNA binding in PNK assays, marking the canonical targeting signal as dispensable for MDH2's RNA-binding activity (Supplemental Fig. S4B,C). Previous studies of yeast mitochondrial MDH (sharing 52.8% identity with the human mitochondrial MDH2) revealed additional internal mitochondrial localization signals functional in the absence of a canonical presequence (Thompson and McAlister-Henn 1989; Small and McAlister-Henn 1997). Furthermore, cryptic targeting sequences had been observed for other yeast mitochondrial proteins like the F1-ATPase β subunit (Bedwell et al. 1987).

**FIGURE 4.**
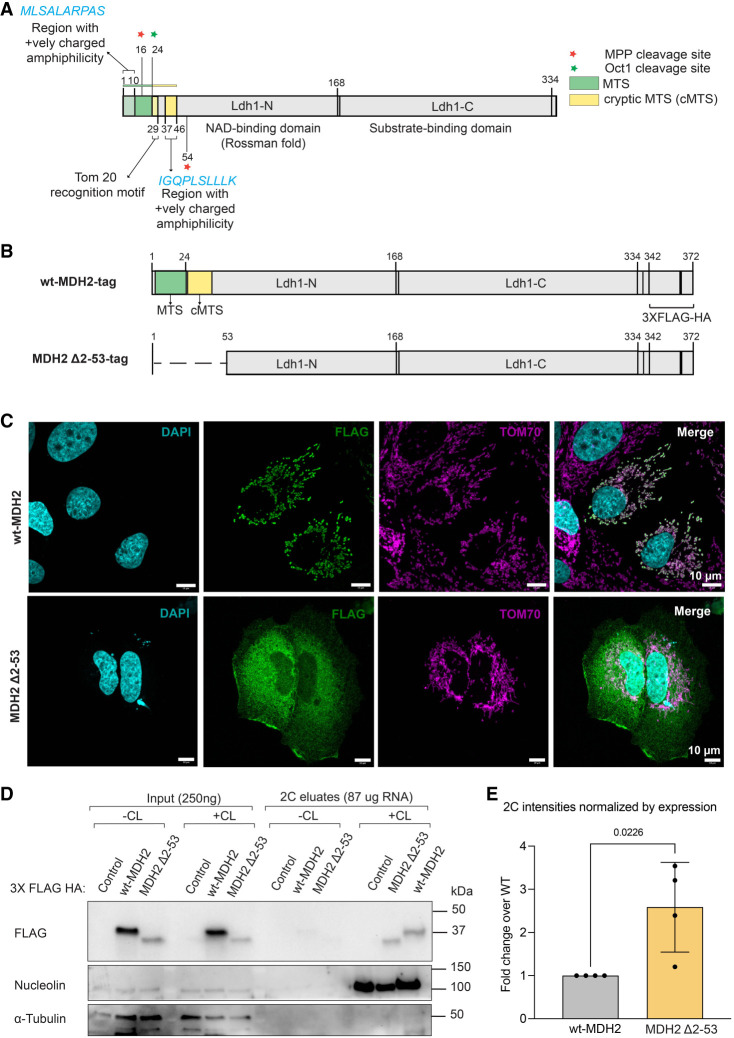
Mitochondrial targeting is dispensable for MDH2's RNA binding. (*A*) Schematic of MDH2 with the sequences relevant for mitochondrial import highlighted. MitoFates software detected the presence of amphipathic α-helices at the N terminus of both wt-MDH2 (highlighted in green as canonical MTS) and MDH2 Δ2–24 (highlighted in yellow as a cryptic MTS). At the N terminus of MDH2 Δ2–24, a Tom20-recognition motif was also detected. (*B*) Schematic of the wt-MDH2 and MDH2 Δ2–53 constructs. (*C*) Confocal immunofluorescence images showing localization of the wt-MDH2 and MDH2 Δ2–53 constructs. Images are maximum intensity projections of *Z*-stack images. (*D*) RNA-binding activity of wt-MDH2 and MDH2 Δ2–53 assessed by 2C experiments. Nucleolin is used as a positive control for RNA binding, and α-tubulin is used as a negative control. (*E*) Relative quantification of the RNA-binding activity of wt-MDH2 and MDH2 Δ2–53 normalized by the expression of the two constructs. Data are reported as mean ± SD, number of experiments *n* = 4, individual *P*-values are indicated according to an unpaired two-tailed *t*-test.

Therefore, we carefully examined MDH2 for additional potential import signals using the MitoFates software (Fukasawa et al. 2015). When the full-length MDH2 protein sequence was used, the expected canonical targeting signal was detected, displaying an amphiphilic α-helical region (Fig. 4A) followed by an MPP (mitochondrial processing peptidase, which generates an intermediate) cleavage site at position 16 and an Oct1 (precursor intermediate peptidase) cleavage site at position 24. When MDH2 Δ2–24 was used as the input for the MitoFates algorithm, a potential cryptic targeting signal was identified within amino acids 25–46, with an additional MPP cleavage site detected at amino acid 54. This sequence region includes a TOM20-recognition motif and a sequence stretch likely to form an amphiphilic α-helix that could assist in the import of MDH2 Δ2–24 (Fig. 4A). Based on this information, we deleted amino acids 2–53, containing all potential import-relevant sequences of MDH2 (Fig. 4B). On analyzing the subcellular localization of MDH2 Δ2–53, we observed diffuse cytoplasmic staining in contrast to the wild-type protein, indicating deficient import into the mitochondria (Fig. 4C).

Next, we expressed wt-MDH2 and MDH2 Δ2–53 in Huh7 cells, and assessed RNA binding by the 2C assay. We observed lower protein expression levels for the cytosolic version of MDH2 compared to the wt-MDH2 (Fig. 4D), possibly because of a lower stability of the protein. Nevertheless, we found that MDH2 Δ2–53 retains similar or even elevated RNA binding (Fig. 4D) compared to wt-MDH2 after normalization for the differences in expression levels (Fig. 4E). This finding reveals that mitochondrial targeting is not required for MDH2's RNA binding. We also conclude that the N-terminal 53 amino acid sequences are dispensable for MDH2's RNA interactions.

### MDH2's RNA binding is sensitive to cellular NAD^+^ levels

**FIGURE 5.**
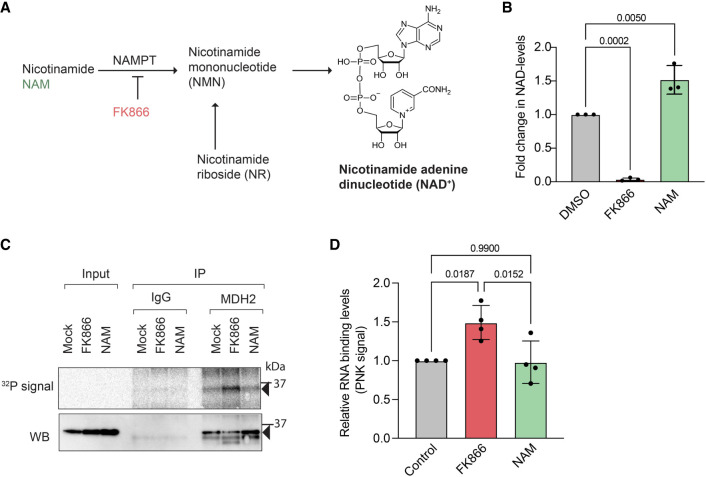
Pharmacological perturbation of cellular NAD^+^ levels affects MDH2's RNA binding. (*A*) Scheme of the salvage synthesis pathway for NAD^+^ showing the steps targeted by NAM and FK866 treatment, respectively. (*B*) Changes in cellular NAD(H) levels were measured after 48 h of treatment. (*C*) PNK assay to assess MDH2's RNA binding on alteration of cellular NAD(H) levels. Black arrows indicate the expected molecular mass of mature MDH2. (*D*) Quantification of RNA binding (PNK signal) of MDH2 after NAM/FK866 treatment. Data are reported as mean ± SD, number of experiments *n* = 3, individual *P*-values as calculated by ordinary one-way ANOVA with Tukey's multiple comparison test correction.

Next, we wanted to explore whether MDH2's RNA binding responds to cellular signals. Because MDH2 binds NAD^+^ as a cofactor, and NAD^+^ levels have been reported to affect the RNA-binding properties of other dehydrogenases (Singh and Green 1993; Sioud and Jespersen 1996; Pioli et al. 2002; Rodriguez-Pascual et al. 2008), we sought to modulate cellular NAD^+^ levels and to monitor the effects on MDH2's RNA-binding activity in cells. FK866, an inhibitor of the rate-limiting step of the cellular salvage NAD^+^ synthesis pathway (Khan et al. 2006; Tan et al. 2013), was used to reduce cellular NAD^+^ cells. We also supplemented cells with the NAD^+^ precursor NAM (Garten et al. 2009) to boost cellular NAD^+^ levels (Fig. 5A,B). Profound reduction of cellular NAD^+^ levels by FK866 treatment significantly activated MDH2's RNA binding (Fig. 5C,D). In contrast, NAM treatment yielding a 1.5-fold increase in cellular NAD^+^ levels did not substantially change MDH2's RNA-binding activity (Fig. 5C,D). The sensitivity of MDH2–RNA interactions to FK866 treatment suggests that NAD^+^ levels may directly or indirectly control MDH2's RNA binding.

## DISCUSSION

In this study, we have characterized the RNA binding of the human mitochondrial enzyme MDH2. We confirmed cellular RNA binding using two orthogonal assays, and identified its RNA interactors by eCLIP. tRNAs, in particular, emerged as top binders, being significantly enriched in IP compared to SMI controls. Whether MDH2 binds full-length tRNAs or tRNA fragments that have been described to be generated especially under conditions of cellular stress (Su et al. 2020; Xie et al. 2020) remains to be defined. MDH2's preference for binding tRNAs is also supported by studies in yeast, which identified yeast MDH2 as an RBP that predominantly binds to small RNAs (Asencio et al. 2023). Interestingly, tRNA binding has also been reported previously for the NAD-dependent dehydrogenase GAPDH (Singh and Green 1993; Nagy and Rigby 1995). Sequence and structure motif analyses on the eCLIP results did not reveal significantly enriched motifs among MDH2's RNA binders. Similar observations were made previously for other RNA-binding metabolic enzymes, like ENO1 (Huppertz et al. 2022).

On investigation of the subcellular location of MDH2–RNA interactions by mitochondrial purification followed by RNA-binding assays, we found that MDH2 binds RNA predominantly outside of mitochondria, which is in excellent accord with the finding that cytosolic RNAs represent the major class of interactors. We also determined that MDH2's RNA binding does not require the N-terminal 52 amino acids of the precursor protein or its mitochondrial localization/targeting. The former implies that the mature protein that reaches the mitochondria bears all amino acids required for RNA binding, although this major MDH2 fraction does not contribute substantially to the overall amount of RNA bound by MDH2 in the conditions we have assayed. The latter observation is in further support of the predominantly cytosolic nature of the MDH2–RNA interaction. We envision that RNA binding by MDH2 may be determined by its environment, which is discussed in more detail below.

Primarily cytosolic RNA binding of a mitochondrial metabolic enzyme is somewhat unexpected, and there are limited data from other systems (Wang et al. 2017). Following viral infection, mitochondrial glutamic-oxaloacetic transaminase (GOT2) binds lncRNA-ACOD1, with an increased fraction of GOT2 appearing in the cytosol. The expression of lncRNA-ACOD1 is induced by viral infection, but it is currently not clear whether the RNA-binding pool of GOT2 is predominantly the precursor form or the mature GOT2 protein present in the cytosol by an as yet-unidentified mechanism. With the exception of proteins like cytochrome *c* that are released into the cytosol from mitochondria during conditions of cellular stress (Garrido et al. 2006), there is little evidence of mature mitochondrial proteins being released back into the cytosol. There have been reports of mitochondrial TCA cycle enzymes (Nagaraj et al. 2017; Kafkia et al. 2022) and the mitochondrial pyruvate dehydrogenase complex being localized to the nucleus (Sutendra et al. 2014). Translocation across the mitochondrial membranes, followed by nuclear import and mitochondria-derived vesicles (Sugiura et al. 2014), has been discussed as a potential mechanism of localization outside the mitochondria. Examination of the MDH2 signal from PNK and 2C assays does not allow a clear assignment of whether the MDH2 preprotein or mature MDH2 binds RNA. The molecular mass difference between the two is only ∼2–3 kDa, and the effect of cross-linked RNA on gel migration must also be taken into account. Therefore, both possibilities remain open for now.

At present, possible functional roles of the MDH2–RNA interactions include both “moonlighting” of MDH2 as a posttranscriptional effector of the fates of the bound RNAs as well as riboregulation of MDH2 function by the bound RNAs (Hentze et al. 2018). Although a “moonlighting” role has been suggested in a previous study (Chen et al. 2017), a rigorous analysis using carefully designed RNA-binding mutants is needed to deconvolute the situation. In addition to previously described effects of RNA on TCA cycle activity by disrupting an FH–MDH2–CS metabolon (Sang et al. 2021), other regulatory effects of RNA on MDH2 can also be imagined, including direct modulation of MDH2's enzymatic activity, regulation of its mitochondrial function by cytoplasmic sequestration, or modulation of mitochondrial import.

Our finding that MDH2–RNA interactions respond to cellular NAD^+^ levels may indicate physiological regulation by the concentration of this cofactor and/or other related metabolites. Nucleotide-binding domains, especially NAD-binding domains, have been associated with RNA binding of metabolic enzymes in several RNA interactome captures (Castello et al. 2015; Perez-Perri et al. 2023). For RNA-binding dehydrogenases like GAPDH (Singh and Green 1993; Sioud and Jespersen 1996; Rodriguez-Pascual et al. 2008) and LDH (Pioli et al. 2002), NAD^+^ has been reported to compete with RNA binding. Thus, NAD^+^ may also compete with RNA to bind MDH2, directly or allosterically, explaining the observed increase in MDH2's RNA-binding following FK866 treatment (Fig. 5). Extending this hypothesis further, compartmentalized NAD^+^ levels in the cytosol and mitochondria could be a determinant of the compartmentalization of MDH2's RNA binding, the typically lower concentration of NAD^+^ in the cytosol being less restrictive to RNA binding than the higher mitochondrial NAD^+^ levels (Di Lisa et al. 2001; Alano et al. 2007; Cambronne et al. 2016; Sallin et al. 2018). However, precise quantification of NAD^+^ levels across compartments is challenging, and there are limited data available on the absolute concentration of NAD^+^ in cytosol compared to the mitochondria (VanLinden et al. 2015). Moreover, MDH2 activity has been found to be regulated by acetylation, a conceivable indirect effect of NAD^+^ modulation via NAD^+^-dependent SIRT deacetylases (Marcus and Andrabi 2018; Parodi-Rullán et al. 2018). Therefore, acetylation-driven regulation of MDH2's RNA binding also deserves to be considered.

In conclusion, our data highlight an unexpected facet of the RNA-binding activity of a mitochondrial enzyme that is not yet functionally understood, but that serves as an example of the scope of RNA–protein interactions in cells. The strong conservation of RNA binding by MDH2 and the effect of altered NAD^+^ levels on RNA binding suggest that the RNA-binding properties of MDH2 warrant further exploration.

## MATERIALS AND METHODS

### Cell culture

Huh7 cells were maintained in a 37°C incubator with 5% CO_2_, with Dulbecco's modified Eagle's medium (DMEM) growth media (reconstituted from Sigma D5523) supplemented with 1 g/L d-glucose (Sigma), 10% heat-inactivated FBS (Thermo Fisher Scientific 10270106), 2 mM l-glutamine (Thermo Fisher Scientific 25030081), and 100 U/mL penicillin-streptomycin (Thermo Fisher Scientific 15140122).

### Protein extracts, SDS-PAGE, and western blotting

For the preparation of cell lysates, cells were washed twice with ice-cold PBS and lysed in RIPA buffer (50 mM Tris/HCl, pH 7.5, 150 mM NaCl, 1% NP40, 0.1% SDS, 0.5% Na-deoxycholate), and then supplemented with fresh complete protease inhibitor cocktail. The lysates were sonicated and cleared by centrifugation at 10,000*g* for 15 min. Protein amounts were estimated with Pierce 660 reagent (Thermo Fisher Scientific 22660) against defined BSA standards (Thermo Fisher Scientific 23208). Samples were prepared for electrophoresis by the addition of 4× loading buffer (Thermo Fisher Scientific NP0007) supplemented with TCEP as a reducing agent and incubated for 10 min at 70°C. 4%–15% Tris-Glycine (Bio-Rad precast gradient gels 5671084) were used for protein separation. Proteins were transferred to PVDF/nitrocellulose membranes using the Transblot semi-dry blotting system (Bio-Rad). The transfer was usually performed at 25 V for 7 min. The membranes were incubated with the blocking buffer (5% milk in 0.05% PBST) for 30 min to 1 h at room temperature (RT). This was followed by incubation with primary antibodies against the target protein at 4°C O/N or 1–3 h at RT on a rotator. The primary antibody was diluted in the blocking buffer. The membrane was washed three times with 0.05% PBST with 5-min incubations. After washing off the unbound primary antibody, the membrane was incubated for 30 min at RT in HRP-conjugated secondary antibody diluted in the blocking buffer. After three washes with 0.05% PBST with 5-min incubations, the membranes were developed in ECL solution (Sigma WBKLS0500) or the SuperSignal West Femto/Atto solutions (Thermo Fisher Scientific A37558, 34095). The antibodies used were rabbit polyclonal MDH2 antibody (Proteintech 15462-1-AP, 1:1000), goat MDH2 antibody (Everest Bio EB13027, 1:1000), FLAG (Sigma-Aldrich F1804, 1:2000) DHX9 (Abcam ab26271, 1:5000), Tom20 (Proteintech 11802-1-AP, 1:1000), Tom70 (Proteintech 14528-1-AP, 1:1000), Nucleolin (Abcam ab50279, 1:1000), and HuR (Proteintech 11910-1-AP, 1:1000).

### PNK assay

Cells (80%–90% confluency) were washed twice with ice-cold PBS and irradiated with UV light (254 nm) of 150 mJ/cm^2^ energy on ice. The cells were lysed in RIPA buffer. The lysates were sonicated and cleared by centrifugation at 10,000*g* for 15 min at 4°C. Around 200–600 μg of total protein lysate was used for PNKs. The lysates were treated with 0.1–0.6 ng/μL RNaseA (Thermo Fisher Scientific EN0531) and 2U of Turbo DNAse (Thermo Fisher Scientific AM2239) for 15 min at 37°C. The lysates were spun down briefly and a fraction of lysate was set aside as input to assess IP efficiency in a western blot. The remaining lysates were for IPs. For IP of MDH2, SureBeads ProteinA magnetic beads (Bio-Rad 1614013) coupled with MDH2 antibody (Proteintech 15462-1-AP) were used. An isotype-matched rabbit IgG (Cell Signalling 02/2018) coupled to SureBeads ProteinA magnetic beads were used in parallel IPs as background control. For IP of overexpressed FLAG-tagged MDH2 variants, FLAG M2 beads (Merck M8823) were used. The lysate-antibody-coupled bead mixture was incubated for 2 h at 4°C . The beads were washed with RIPA buffer (50 mM Tris/HCl, pH 7.5, 150 mM NaCl, 1% NP40, 0.1% SDS, 0.5% Na-deoxycholate, fresh complete protease inhibitor cocktail), RIPA-HS buffer (50 mM Tris/HCl, pH 7.5, 500 mM NaCl, 1% NP40, 0.1% SDS, 0.5% Na-deoxycholate, fresh complete protease inhibitor cocktail), and PNK buffer (50 mM Tris/HCl, pH 7.5, 50 mM NaCl, 0.5% NP40, 10mM MgCl_2_, fresh 5 mM DTT, fresh complete protease inhibitor cocktail) for 2 min each. For labeling the RNA, the beads were incubated in the 30 μL T4 PNK reaction mix (0.3 μL of 0.1 μCi/μL γ-32P ATP, 3U of T4 PNK enzyme [NEB M0201L] in 1× PNK reaction buffer containing 1 mM DTT) for 15 min at 37°C. Following four washes with the PNK buffer, proteins were eluted off the beads at low pH (0.1 M glycine, pH 2.0) for endogenous MDH2 IP and incubation with 250 μg/mL 3XFLAG peptide (Merck F3290) in the case of FLAG IP. When eluted at low pH, the sample solution was neutralized by the addition of 1.5 M Tris–HCl, pH 8.5. After the addition of 4× sample loading buffer (Thermo Ficher Scientific NP0007) with added TCEP (Merck 646547), the samples were boiled for 10 min at 70°C. The samples were resolved using SDS-PAGE and further transferred to a nitrocellulose membrane. After drying the membrane, it was exposed to a phosphor screen overnight. The screen was scanned on a Typhoon FLA 9500 system at 600 nm. The membrane was further immunoblotted with MDH2 antibody to estimate IP efficiency.

### 2C Assay

2C was performed as described (Asencio et al. 2018). Huh7 cells were grown to 80%–90% confluency in 15-cm dishes. Cells were washed twice with ice-cold PBS and irradiated with UV light (254 nm) of 150 mJ/cm^2^ energy on ice. The cells were lysed in RIPA buffer (50 mM Tris/HCl, pH 7.5, 150 mM NaCl, 1% NP40, 0.1% SDS, 0.5% Na-deoxycholate, fresh complete protease inhibitor cocktail). The lysates were sonicated and cleared by centrifugation at 10,000*g* for 15 min; 1–1.5 mg of samples were processed for 2C. A fraction of the input was set aside for western blot assessment. Samples were processed according to the manual using the components of the Zymo RNA mini kit (Zymo R1013). The RNA and RNA–protein complexes were eluted from the column with 100 µL Nuclease-free water. The eluates were treated with 5U Turbo DNAse (Thermo Fisher Scientific AM2239) for 30 min at 37°C. In selected samples, 5 U RNase I (Ambion AM2294) was included in the reaction. The samples were then processed for a second round of 2C as described above. The eluates were treated with RNase I (Ambion AM2294) (with exceptions) to digest the eluted RNA in the RNP complexes and visualize the protein around the expected molecular mass using western blotting. Samples were mixed with 4× sample loading buffer (Thermo Fisher Scientific NP0007) supplemented with TCEP and incubated for 10 min at 70°C before gel loading. The samples were resolved using SDS-PAGE and immunoblotted with the antibodies of interest.

### eCLIP

eCLIP was performed according to published protocols (Van Nostrand et al. 2016) with the following changes. Seventy-five microliters of SureBeads ProteinA magnetic beads (Bio-Rad 1614013) was coupled with 6 μg of MDH2 antibody (Proteintech 15462-1-AP) for 2 h at 4°C. Five hundred micrograms of precleared cell protein lysate was treated with 1 U RNase I (Ambion AM2294) for 5 min at 37°C. After the addition of 4 μL of RNAsin Plus ribonuclease inhibitor (Promega N2611), the samples were spun down, transferred to a new tube, and used for IP with the antibody-coupled beads. Before IP, 1% of the input was set aside as SMI control. The IP was performed at 4°C for 2 h. Isotype-matched IgG was used as a control. The RNA adaptors X1A (/5Phos/rArUrArUrArGrGrNrNrNrNrNrArGrArUrCrGrGrArArGrArGrCrGrUrCrGrUrCrUrArG/3SpC3/), RiL19 (/5Phos/rArGrArUrCrGrGrArArGrArGrCrGrUrCrGrUrG/3SpC3) and DNA adaptors AR17 (ACACGACGCTCTTCCGA) and (rand3Tr3/5Phos/NNNNNNNNNNAGATCGGAAGAGCACACGTCTG/3SpC3/) were used for reverse transcription and linker ligation, respectively, as described in the original eCLIP protocol (Van Nostrand et al. 2016). Following library preparation till the cDNA step as described previously (Van Nostrand et al. 2016), we optimized the number of cycles required for the final PCR using test PCRs with 10% of the cDNA sample followed by gel analysis (6% TBE gels, Thermo Fisher Scientific EC6265BOX). cDNA libraries from MDH2 IP (14 cycles), IgG IP (14 cycles), and SMI (nine cycles) were multiplexed and analyzed with paired-end sequencing on NextSeq 500 using different cycle numbers for read 1 (20 cycles) and read 2 (130 cycles), with index 1 and 2 read by eight cycles each.

### eCLIP data analysis

The quality of the eCLIP raw reads was examined using fastqc (Andrews 2010) (v0.11.8). The Unique molecular identifier (UMI) barcodes, attached during library preparation, were appended to the read name using UMI tools (v1.0.0) extract (Smith et al. 2017). The adapters were trimmed and shorter reads (length <18 nt) were discarded using cutadapt (v2.5) (Martin 2011). The trimmed reads were mapped to the human genome (GRCh38.v23 from GENCODE) using STAR (v2.7.1a) (Dobin et al. 2013). Reads that align to more than 10 genomic locations were discarded using “outFilterMultimapNmax 10” parameter. The other STAR parameters used are as follows: “sjdbOverhang 149,” “outSAMunmapped Within,” “outFilterMultimapScoreRange 1,” “outFilterType BySJout,” “outReadsUnmapped Fastx,” “outFilterScoreMin 10,” “alignEndsType Extend5pOfRead1,” “genomeLoad NoSharedMemory,” “outSAMattributes All,” “outFilterMismatchNoverLmax 0.08,” “seedSearchStartLmax 10.” All others are set to default. PCR duplicated reads from the aligned reads were removed using UMI tools dedup based on the UMI barcode.

The tRNAs, provided by tRNAscan (Lowe and Chan 2016), were added to the GENCODE (v23) annotation of the GRCh38.v23 genome and preprocessed with the htseq-clip suite (Sahadevan et al. 2022). These annotations were reformatted into sliding windows of 50 nt with a step size of 20 nt, using htseq-clip annotation and createSlidingWindows functions. The cross-link site was extracted as 1 nt upstream of the alignment start position for each aligned read using the htseq-clip extract function. htseq-clip count was used to quantify the total number of cross-link sites per sliding window and was converted into an R-friendly count matrix using htseq-clip createMatrix function. The count matrix was prefiltered to retain only those windows with a minimum of 10 cross-link sites in at least three samples. DEWSeq (v1.0.0) (Schwarzl et al. 2024), a R/Bioconductor package, was used to determine significant cross-link enrichment of windows in IP samples over the SMI control samples (*P*_adj_ ≤ 0.05 and log_2_FC ≥ 1). The Bonferroni method (Bland and Altman 1995) was used to control the familywise error rate, and the Benjamini–Hochberg method (Benjamini and Hochberg 1995) was used to control the false discovery rate. Windows with significant enrichment in MDH2 IP in comparison to SMI (*P*_adj_ ≤ 0.05 and log_2_FC ≥ 1) were selected for further downstream analysis and overlapping windows among these were merged into binding regions. These binding regions (782) were then screened to remove those binding sites with zero read counts in the input samples (to prevent artificial enrichment), as well as aberrant single-peak cross-linking sites (mostly in intronic regions). To generate Figure 2A, the number of cross-linking counts of MDH2 on the identified regions (aggregated normalized counts of cross-link sites across all windows in the region) was used (Supplemental Table S1).

### Protein expression and purification

petM22 protein purification constructs were transformed into BL21 Rosetta (DE3) cells. The transformed bacteria were grown overnight at 37°C in 25 mL Lysogeny broth (LB) media supplemented with Kanamycin (Sigma K0254). For autoinduction of MDH2 and eGFP proteins, the preculture was diluted 1:100 in Terrific broth (TB) supplemented with 1.5% lactose, 0.05% glucose, 2 mM MgSO_4_ for growth at 37°C until the OD reached 0.7–0.8. The bacterial culture was then transferred to an 18°C incubator for overnight growth with shaking at 180 rpm. The Strep-Tactin Superflow high-capacity resin (IBA Lifesciences 2-1208-002) was used to purify the protein. First, the cells were lysed by sonication in 100 mM Tris/HCl, pH 8.0, 150 mM NaCl, 1 mM EDTA supplemented with free protease inhibitors, and 250 μg/mL lysozyme. The cells were sonicated for 7 min at 50% duty, 70% power on wet ice, and centrifuged at 15,000*g* for 15 min to remove debris. The supernatant was allowed to flow through a polypropylene column packed with Strep-Tactin resin. The column was washed five times with 100 mM Tris/HCl, pH 8.0, and 150 mM NaCl. The column-bound protein was eluted in fractions with 100 mM Tris/HCl, pH 8.0, 150 mM NaCl, 1 mM EDTA, and 2.5 mM desthiobiotin (IBA Lifesciences 2-1000-002). The eluted fractions containing the highest concentration of the expressed protein were pooled and dialyzed against the storage buffer containing 10 mM Tris/HCl, 150 mM NaCl, and 5% glycerol. The sample concentration was estimated by UV–Vis spectroscopy (absorbance at 280 nm) and using defined BSA standards (Thermo Fisher Scientific 23208) through SDS-PAGE analysis. If needed, the samples were concentrated using Amicon Ultra-0.5 Centrifugal Filter Unit (Millipore UFC5010). The purified protein aliquots were stored at −80°C.

### Electromobility shift experiments

Binding reactions containing 10 nM Cy5-labeled RNAs (IDT, Sigma) and defined amounts of proteins were set up in 10 mM Tris–HCl pH 7.5, 150 mM NaCl, 20% glycerol, 0.5 mM DTT, 5 mM MgCl2, 0.01 μg/µL BSA and incubated at 25°C for 20 min. The probe sequences for the Arg TCT 3-2 tRNA and the control probe were, respectively, GGCUCUGUGGCGCAAUGGAUAGCGCAUUGG and AUAUUUAGCUCAGGCCCGUGGCGCC. The binding reactions were resolved and analyzed using native polyacrylamide gel electrophoresis (4%–15% Tris-Glycine gradient gels in running buffer containing 34 mM Tris–HCl, 66 mM HEPES, 0.1 mM EDTA), with the separation done at 100 V for 75 min. Fluorescent signal on the RNA was analyzed using Typhoon FLA-9500.

### Mitochondrial fractionation and cross-linking

Cells grown to 80%–90% confluency were washed two times with ice-cold PBS and harvested in 3 mL of 20 mM HEPES pH 7.4–7.6, 220 mM mannitol, 70 mM sucrose, 1 mM EDTA supplemented with 2 mg/mL BSA, complete EDTA-free protease inhibitors, and RNAsin (Promega N2611). Cells were allowed to swell by incubation for 10 min at 4°C and lysed with 20–25 strokes of a Dounce homogenizer. Nuclei were pelleted by centrifugation at 1000*g* for 10 min at 4°C. The supernatant was centrifuged at 15,000*g* for 20 min at 4°C to pellet mitochondria. The mitochondrial pellet was washed two times with 20 mM HEPES pH 7.4–7.6, 220 mM mannitol, 70 mM sucrose, and 1 mM EDTA. The wash buffer was removed by centrifugation for 20 min at 4°C. The pellet was resuspended in 100 μL of 20 mM HEPES pH 7.4–7.6, 220 mM mannitol, 70 mM sucrose, and 1 mM EDTA, and then irradiated with UV light after spreading on a glass bottom dish. The mitochondria were lysed by sonication after adding the excess volume of RIPA buffer (50 mM Tris/HCl, pH 7.5, 150 mM NaCl, 1% NP40, 0.1% SDS, 0.5% Na-deoxycholate, fresh complete protease inhibitor cocktail). The lysates were snap-frozen and stored at −80°C till further use. Whole-cell samples were processed at the same time by cross-linking and lysis in RIPA buffer (50 mM Tris/HCl, pH 7.5, 150 mM NaCl, 1% NP40, 0.1% SDS, 0.5% Na-deoxycholate, fresh complete protease inhibitor cocktail).

### Transfections

Cells were transfected using either the forward (plating cells on the day before) or reverse method (plating cells right before) transfection. Lipofectamine 3000 reagent (Thermo Fisher Scientific 11668030) was used according to the manufacturer's instructions to transfect plasmids.

### Immunofluorescence

Cells were grown to 60% confluency on coverslips/multiwell glass bottom dishes (ibidi) and fixed with 4% paraformaldehyde (Thermo Fisher Scientific 28906). 0.2% Triton X-100 was used to permeabilize cells by incubation at RT for 10 min. Samples were blocked in the IF blocking buffer (2.5% BSA in 0.05% Triton X-100) for 30 min at RT. After blocking, samples were incubated with antibodies diluted in IF blocking buffer for 1 h at 37°C. Samples were washed three times in 0.05% Triton X-100 before incubating with the secondary antibody labeled with the fluorescent dye (also diluted in the blocking buffer) for 45 min to 1 h at RT in the dark. The following secondary antibodies from Life Technologies were used: antirabbit Alexa Fluor 488 (A21206), antimouse Alexa Fluor 488 (A21202), and antirabbit Alexa Fluor 647 (A21245). Nuclei were stained by incubation with Nucblue reagent (Thermo Fisher Scientific R37606) diluted in 0.05% Triton X-100 (Invitrogen) for 10 min. The samples were further washed two times before mounting on a glass slide using ProLong Diamond antifade mountant (Invitrogen P36961) or the addition of mounting solution (ibidi 50001) in glass dishes. The glass slides were left to cure overnight at RT before imaging and stored for the long term at 4°C. Images were acquired on an Olympus 3000FV microscope with either a 60× objective/40× objective.

### Cellular NAD modulation

Cells growing at ∼60% confluency were treated with 5 nM FK866 (Merck 481908), 5 mM NAM (Sigma N0636), or DMSO (Merck 1.02950) for 48 h before harvest. A fraction of the cell lysates was used to estimate NAD(H) levels using the NAD/NADH quantification kit (Sigma MAK037) according to the manufacturer's instructions.

### Quantification and statistical analysis

GraphPad Prism version 9.3.1 was used for statistical analysis of experimental data (excluding sequencing data). The tests used are documented in the figure legends wherever applicable.

## DATA DEPOSITION

Sequencing data have been submitted to GEO and are available under accession number GSE249145.

## SUPPLEMENTAL MATERIAL

Supplemental material is available for this article.

## Figures and Tables

**Michelle Noble:**
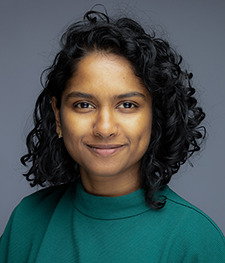


## MEET THE FIRST AUTHOR

***Meet the First Author(s)* is an editorial feature within *RNA*, in which the first author(s) of research-based papers in each issue have the opportunity to introduce themselves and their work to readers of *RNA* and the RNA research community. Michelle Noble is the first author of this paper, “Cytosolic RNA binding of the mitochondrial TCA cycle enzyme malate dehydrogenase.” Michelle was a graduate student in the laboratory of Professor Dr. Matthias Hentze at EMBL Heidelberg, where she finished her PhD in 2023. The focus of her PhD was on characterizing the RNA-binding activity of mitochondrial metabolic enzymes.**

**What are the major results described in your paper and how do they impact this branch of the field?**

In this paper, we focused on understanding the RNA-binding ability of a mitochondrial metabolic enzyme, malate dehydrogenase 2. We validated MDH2–RNA interactions in Huh7 cells and identified MDH2's RNA-binding partners using eCLIP. Given the cytosolic origin of RNAs bound by MDH2, we looked at the subcellular locale of these interactions and found it to be predominantly outside of the mitochondria. Moreover, we found that the RNA-binding ability of MDH2 increased in conditions of decreased cellular NAD^+^ (MDH2's cofactor for its enzymatic function) levels, indicating that the MDH2–RNA interactions might be regulated.

**What led you to study RNA or this aspect of RNA science?**

I had worked in the laboratory of the late Professor Dr. Elisa Izaurralde, where I discovered a whole new world of RNA and its regulators. I chose to continue my journey into the RNA field in Matthias's laboratory. The laboratory is currently exploring noncanonical RNA-binding proteins, particularly metabolic enzymes identified to bind RNA. I followed that route to explore MDH2's RNA-binding function.

**During the course of these experiments, were there any surprising results or particular difficulties that altered your thinking and subsequent focus?**

It was particularly surprising to see that the majority of RNAs bound by MDH2 were cytosolic. For a protein where probably >90% of the protein is in the mitochondria, a tiny cytosolic fraction of MDH2 seems to show most of the RNA-binding function. It would have been wonderful to follow up on the mechanisms and regulation of MDH2–RNA interactions, but it was technically very challenging to study the compartment-specific RNA binding of MDH2 in detail.

**If you were able to give one piece of advice to your younger self, what would that be?**

Ask for help as much as you can. It can be for discussing ideas, troubleshooting problems, or just when you are feeling overwhelmed. Science progresses faster when we collaborate and support each other.

**Are there specific individuals or groups who have influenced your philosophy or approach to science?**

I am immensely grateful to have had both Elisa and Matthias to guide me into the RNA world. I spent a lot of my PhD time working together with Dr. Aindrila Chatterjee. It was filled with great scientific discussions, and Aindrila's energy to discover and test new ideas was very inspiring. I am very grateful also to have had very supportive laboratory members who taught me a lot.

**What are your subsequent near- or long-term career plans?**

I have recently started as a postdoctoral scientist at Bayer in Germany, where I will be continuing to work on RNA.
